# Case Report: Fatal cytomegalovirus pneumonia after CAR-T cell therapy in the long-term follow-up

**DOI:** 10.3389/fimmu.2023.1226148

**Published:** 2023-10-02

**Authors:** Jiali Cheng, Jin Huang, Wenyue Cao, Liang Huang, Xia Mao, Liting Chen, Jianfeng Zhou, Na Wang

**Affiliations:** Department of Hematology, Tongji Hospital, Tongji Medical College, Huazhong University of Science and Technology, Wuhan, Hubei, China

**Keywords:** case report, CMV disease, CMV pneumonia, CAR-T cell therapy, B-ALL

## Abstract

**Introduction:**

The rapidly developed CAR-T cell therapy has a unique profile of side effects, which perhaps has not been totally realized and understood, especially the late-phase toxicity. CMV is prevalent world-wide and establishes a life-long latency infection. It can lead to life-threatening complications in immunocompromised host, and little is known about CMV disease in patients after CAR-T cell therapy. Here, we report a patient who developed possible CMV-pneumonia three months after anti-CD19 and anti-CD22 CAR-T cell therapy for relapsed B-ALL, contributing to the understanding of severe side-effects mediated by virus infection or reactivation in patients receiving CAR-T cell infusion.

**Case presentation:**

A 21-year old male patient with relapsed B-ALL received anti-CD19/22 CAR-T cell therapy, and achieved complete remission 2 weeks after the infusion. However, three months later, the patient was hospitalized again with a 10-day history of fever and cough and a 3-day history of palpitations and chest tightness. He was diagnosed with possible CMV pneumonia. Under treatment with antiviral medicine (ganciclovir/penciclovir), intravenous gamma globulin and methylprednisolone and the use of BiPAP ventilator, his symptoms improved, but after removing penciclovir his symptoms went out of control, and the patient died of respiratory failure 22 days after admission.

**Conclusion:**

CMV infection/reactivation can occur in patients long after receiving anti-CD19/22 CAR-T cell therapy, and induce fatal pneumonia, which reminds us of the late side effects associated with immunosuppression after CAR-T cell infusion.

## Background

Tumor immunotherapy has rapidly developed, and chimeric antigen receptor (CAR)-T cell therapy for B cell malignancies is a good example. The extraordinary outcomes of anti-CD19 CAR-T therapy for relapsed or refractory non-Hodgkin B cell lymphoma (B-NHL) (40%-60% complete remission, CR) and B cell acute lymphoblastic leukemia (B-ALL) patients (70%-94% CR) have led to the U.S. food and drug administration (FDA) to grant the first approval for cellular therapy (1–3). The synthetic CAR endows T cells with the capability of recognizing and cytolyzing target cells expressing the specified antigen (4). Moreover, it is a “live” drug, which expands and persists *in vivo*, providing long-term disease surveillance, and, therefore, the related side effects may also happen late after CAR-T infusion. The well-noticed toxicities are cytokine-release syndrome (CRS) and neurotoxicity, generally occurring within one month after infusion. Infectious complications within 3 months after infusion have recently been seen, including hepatitis B virus (HBV) and cytomegalovirus (CMV) reactivation/infection (5, 6). Whilst the risk of viral reactivation/infection still exists during the late-phase (three months post-infusion) of CAR-T cell therapy due to the persistent immune suppression, related reports are limited.

CMV, a linear double-stranded DNA virus, belongs to the beta-herpesvirus subfamily, and is prevalent among the population, with over 95% seropositivity in Chinese adults (7). It usually results in an asymptomatic latent infection (the viral DNA is undetectable in blood or other body fluids) in immunocompetent individuals. CMV infection (reactivation) is diagnosed based on the detection of CMV DNA or antigen in body fluids or isolation of CMV in tissue specimens (in a previous seropositive individual) (8). CMV disease includes CMV syndrome, and end-organ disease that is defined as presence of the clinical symptoms and identification of CMV in the relevant tissues. Notably, CMV DNA detected in the blood, together with clinical symptoms, is insufficient to diagnose a proven CMV disease (except for CMV retinitis), but support the possibility of other CMV diseases when other causations for the symptoms are excluded and anti-CMV therapy works (8). Even in immunocompromised patients, not all CMV infection/reactivation develops into CMV disease. To date, CMV infection/reactivation has mainly been studied in patients undergoing hematopoietic stem cell transplantation (HSCT) or organ transplantation, and in patients infected with human immunodeficiency virus (HIV) (9, 10). The incidence rates ranged from 17% to 92%, with distinct manifestations, including myelosuppression, pneumonia, gastrointestinal symptoms, disorders of the central nervous system and even myocarditis (11, 12). It is consistent with the literature that a deficiency in cellular immunity often results in CMV infection/reactivation and diseases. However, a few studies, including case reports and retrospective studies, have shown that CMV infection/reactivation may occur in patients receiving rituximab therapy, which eliminates B cells (13).

Here, we report a case of fatal CMV pneumonia three months after anti-CD19/22 CAR-T cell therapy. This case may raise awareness of long-term risk of severe viral infection in the era of anti-CD19/22 CAR-T cell therapy.

## Case description

A 21-year-old man was diagnosed with B-ALL 5 years ago (August 2015) and received chemotherapy consisting of one cycle of VCDLP (CTX, vindesine, daunorubicin, prednisone, PEG-aspargase) for induction (14)), and one cycle of MTX plus PEG-aspargase for consolidate (14). After these two cycles of therapy, minimal residue disease (MRD) was still positive. Therefore, he underwent a second-line therapy with MA (MTX and Arac) (15), and reached MRD-negative complete remission (CR). In March 2016, he received allo-HSCT (allogenic hematopoietic stem cell transplantation) from an HLA-matched unrelated donor, and all went well with no occurrence of CMV reactivation. Four months later, a disease relapse was suspected, and the patient was admitted to our hospital for further treatment, where he underwent bone marrow (BM) examination. Flow cytometry revealed 18.5% abnormal B lymphoblasts, and immunohistochemistry showed significant hyperplasia of abnormal lymphoblasts with CD34^+^ TdT^+^ CD79a^+^ CD10^+^ CD19^+^ CD22^+^ CD3^-^ BCL2^+^ MPO^-^ and Ki-67 Li 90%. Gene examination revealed a missense mutation in the *NOTCH2* gene and a splice variant of the *IKZF1* gene. No evidence of central nervous system (CNS) invasion or any other extramedullary diseases were identified. Serological tests were negative for HBV antigens/antibodies except for HBsAb, but they were not performed for CMV antibodies. No CMV DNA or HBV DNA was detected in the blood. The chest CT scan and hematobiochemical results were consistently normal. He was allergic to ofloxacin and had no history of exposure to HBV, HIV, tuberculosis, or any other infectious diseases. No other clinical history, familiar or psycho-social history of importance.

**Figure 1:**
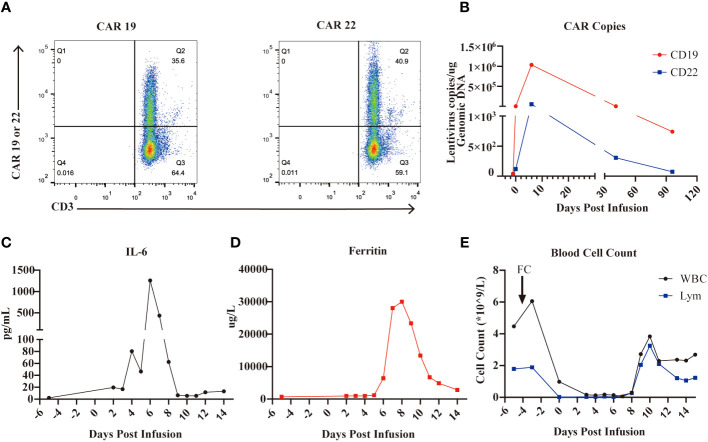
CD19 and CD22 CAR-T cell cocktail therapy for B-ALL. **(A)**, The CAR19 and CAR 22 transfection rate determined by flow cytometry are 35.6% and 40.9%, respectively. **(B)**, The expansion of CAR-T cells in the PB measured by the CAR-contained lentivirus copies. **(C)**, Levels of IL-6 during CAR-T cell therapy. **(D)**, Levels of ferritin during CAR-T cell therapy. **(E)**, Numbers of WBC and lymphocyte during CAR- T cell therapy.

Given the relapse after HSCT, we treated the patient with the sequential infusion of anti-CD19 and anti-CD22 CAR-T cells (ChiCTR-OPN-16008526). It took two weeks to manufacture the patient’s own anti-CD19 and anti-CD22 CAR-T cells from collected peripheral blood mononuclear cells (PBMCs). The transfection rates of CD19 CAR and CD22 CAR were 35.6% and 40.9%, respectively (**Figure 1A**). The patient was treated with 3-day conditioning chemotherapy consisting of fludarabine 25 mg/m^2^ and cyclophosphamide 20 mg/kg per day, and was sequentially transfused with CD19 CAR-T cells and CD22 CAR-T cells at doses of 1.5x10^6^ cells/kg and 1x10^6^ cells/kg, respectively. As shown in **Figure 1B**, CAR-T cells expanded well *in vivo* with CD19 and CD22 CAR copy numbers of 1,034,286 and 52,857.14 per µg genomic DNA, respectively, on day 6. Meanwhile, after the infusion of CAR-T cells, the patient developed grade 3 CRS manifested as high fever (max temperature of 41°C), hypotension (lowest at 88/42 mmHg, responsive to fluids therapy), hypoxia (requiring high-flow nasal cannula oxygen), coagulopathy (requiring fresh frozen plasma), pulmonary edema characterized by extensive rales among both lower lobes of the lung, and grade 3 immune effector cell-associated neurotoxicity syndrome (ICANS) with two-side blurred vision. Eye examinations found patchy bleeding around the infratemporal branch of the central retinal vein and scattered exudation in the other parts of the right retina (suspected to be associated with coagulopathy and severe thrombocytopenia), and mild retinal edema (no hemorrhage was observed) in the left eye. Laboratory testing revealed markedly increased levels of IL-6 and ferritin after CAR-T cell infusion, as shown in **Figures 1C, D**, with peak values of 1,260 pg/mL and 30,014 µg/L on day 6 and day 8, respectively. The serum levels of aspartate aminotransferase (AST), alanine aminotransferase (ALT), N-terminal B-type natriuretic peptide (NT-proBNP), and hypersensitive troponin were also abnormally elevated. After one session of plasma exchanges [to selectively eliminate inflammatory cytokines (16)], two 40-mg doses of methylprednisolone, and other supportive treatments, the inflammatory cytokine storm was gradually controlled, with both the levels of IL-6 and ferritin falling to baseline levels 2 weeks after the infusion as well as the levels of AST, ALT, NT-proBNP, and hypersensitive troponin. The blurred vision disappeared spontaneously two days later. In addition, the patient experienced severe myelosuppression but recovered two weeks later, as depicted in **Figure 1E**. The patient received prophylactic anti-infective treatment (excluding antiviral therapy) after CAR-T cell infusion, and no severe infection was observed during treatment. BM aspiration was performed on day 14, indicative of complete remission by flow cytometry.

**Figure 2:**
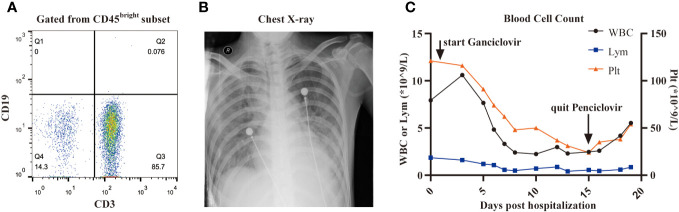
CMV reactivation. **(A)**, B cells in peripheral blood determined by flow cytometry is 0% three months after CAR-T cell infusion. **(B)**, The image of chest X-ray showed prominent interstitial infiltration. **(C)**, The dynamic changes of the number of WBC, lymphocyte and platelet after the use of ganciclovir or penciclovir.

**Figure 3:**
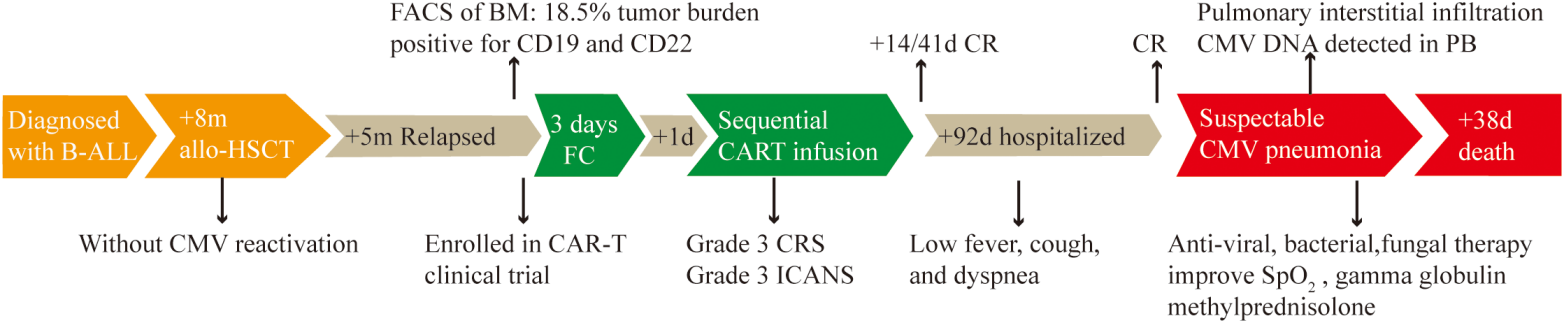
Timeline of disease progression and relevant treatment. B-ALL, acute B-cell lymphoblastic leukemia. +8m/+5m: 8/5 months later. Allo-HSCT: allogeneic hematopoietic stem cell transplantation. FACS: Fluorescence activated Cell Sorting. BM: bone marrow. FC: fludarabine (25 mg/m^2^) and cyclophosphamide (20 mg/kg per day). +1d: 1 day later. The same principle for +14d, +41d, +92d and +38d. CRS, cytokine release syndrome; ICANS, Immune effector cell-associated neurotoxicity syndrome; CR, complete remission; PB, peripheral blood.

On December 3, three months after CAR-T cell treatment, the patient was hospitalized with a 10-day history of low fever (37-38 °C) and cough, and a 3-day history of palpitations, chest tightness and dyspnea. The primary disease was well controlled, and B cells were still absent (**Figure 2A**). The findings on chest X-ray suggested pulmonary infection and interstitial infiltration (**Figure 2B**). CMV DNA, rather than bacterial DNA or fungal DNA, was detected in the peripheral blood by next-generation sequencing (NGS) sequencing. Given the patient’s previous medical history, clinical manifestations, and laboratory and radiographic evidence, he was diagnosed with suspectable CMV pneumonia (fiberoptic bronchoscopy was contraindicated as the patient was oxygen dependent, and therefore, no pneumonia tissue was available to confirm CMV infection). On December 4, the patient received antiviral (ganciclovir 5mg/kg q12h) therapy, as well as preventive anti-bacteria (tigecycline 50mg q12h, and sequential use of cefoperazone/sulbactam 3g q8h, meropenem 0.5g q6h and imipenem/cilastatin 1g q8h) and anti-fungi (sequential use of voriconazole 0.2g q12h and caspofungin 50mg qd) therapy (all intravenously). However, the symptoms worsened rather than alleviated, with the occurrence of diarrhea (yellow watery stool) and an SpO2 of 90% despite using oxygen mask on December 7. Therefore, we introduced intravenous gamma globulin 20 g/day, methylprednisolone 40 mg/day and bilevel positive airway pressure (BiPAP) ventilation on December 8, after which his pulmonary symptoms gradually improved. Ganciclovir was changed to penciclovir (5mg/kg q12h, intravenous drip) due to the side effects of limb numbness, chills and pain at the infusion site. During treatment with these two antiviral drugs, the patient’s WBC count and platelet count decreased progressively (**Figure 2C**). We stopped penciclovir on December 18 when the clinical symptoms had improved. Meanwhile, CMV DNA was negative in the blood, and a chest X-ray showed reduced inflammatory filtration. However, the side effects of myelosuppression aggravated, and three days after treatment discontinuation with penciclovir, the patient again reported chest tightness and shortness of breath. We reintroduced penciclovir on December 21, but the patient’s symptoms gradually worsened. He died on December 25 from respiratory failure. The key events in this case are summarized in **Figure 3**.

## Discussion

Even though the conditions for the diagnosis of possible CMV pneumonia were not fully met in this case, the new presence of CMV DNA in the blood three months after CAR-T infusion and the rapidly progressing clinical course should make us aware of paying special attention to late-phase toxicity, while encouraged by the impressive response of CAR-T therapy. Here we would like to discuss the following questions: 1) the rate of CMV infection/reactivation during CAR-T cell therapy and the clinical significance; 2) the possible mechanisms and high-risk factors related to CMV infection/reactivation, especially in CAR-T cell therapy; 3) the effectiveness of prevention strategies and the advancement in therapies.

First, the infection/reactivation of CMV after CAR-T cell therapy was rarely reported. The available information includes: four cases of CMV DNAemia with one developing CMV pneumonia (not proven) in a 133-patient cohort within 90 days post anti-CD19 CAR-T infusion (5); three cases of detectable viral DNA with no significant symptoms out of 60 cases within one year after therapy (17); no CMV reactivation in 83 (within 90 days after therapy)/53 (within 180 days after therapy) cases of cohorts reported by Vora et al. and Park et al. (18, 19). In allo-HSCT, the incidence of CMV infection ranges from 30% to 70%, with a CMV disease rate of 10-40%. Approximately 70% of the patients who developed CMV disease died (20). However, CMV DNA is routinely monitored in allo-HSCT recipients but not in CAR-T recipients, and CMV infection/reactivation can be asymptomatic. Meanwhile, approximately 31% of CMV reactivation were coinfected with other microbes (21), which may lead to the neglection of CMV infection in the early stages. Moreover, CAR-T recipients are typically heavily pre-treated, with around one third experiencing HSCT, which may lead to an increased risk of CMV infection/reactivation (discussed in the following part). Therefore, the rates of CMV infection/reactivation after CAR-T therapy and its clinical significance are possibly underestimated.

Second, CMV infection/reactivation mainly occurs in immunocompromised patients, especially with cellular deficiency, like HIV-positive patients and organ transplant patients. It is well established that CMV-specific CD4^+^ T cells and CD8^+^ T cells play a pivotal role in controlling the replication and reactivation of CMV through a cytotoxic mechanism, like the secretion of IFN-γ (22). A recent study showed that humoral immunity also plays a key role in inhibiting CMV infection/reactivation. Injection of strain-specific protective antibodies prevented CMV reactivation by restricting the dissemination of CMV in mice with no plasma cell (23).

In the setting of anti-CD19/22 CAR-T therapy, the impaired immune functions caused by multiple lines of chemotherapy (24), fludarabine-containing conditioning chemotherapy (11, 25), and CAR-T cell activity-induced B cell aplasia potentially increase the risk of CMV infection/reactivation. The situation is more complicated for patients who have relapsed after allo-HSCT. Risk factors of CMV infection/reactivation in HSCT recipient are detailly summarized (26). Nevertheless, 90% of CMV infection/reactivation occurred within 100 days post HSCT (27), indicative of the minimal possibility of the infection in this case secondary to HSCT (happened 7 months after HSCT). Generally, risk factors for CMV infection/reactivation and CMV disease in CAR-T cell therapy are scarcely understood, which may be due to the limited cases reported. In this case, the complete blood count (CBC) was already recovered when CMV reactivation happened, but the immune functions were unclear. More basic studies and clinical data are needed to further evaluate the hematological toxicity of CAR-T therapy (not only the CBC, but also the immune function) and associate it with CMV infection/reactivation.

Finally, there are no available guidelines for the prevention of CMV infection/reactivation in CAR-T therapy. Experience in allo-HSCT settings may provide a possible reference. Before conduction of HSCT, serological status for CMV will be determined (unknown in this case regretfully), guiding the infusion of blood products. A seronegative recipient should avoid transfusion of seropositive blood, unless leukocyte-depleted (28). Even though reported to reduce the risk of CMV infection/reactivation (acyclovir and valaciclovir) (29) and CMV disease (ganciclovir), prophylactic anti-CMV therapy using acyclovir, valaciclovir, or ganciclovir, does not improve the overall survival (30). Additionally, preventive anti-CMV therapy increases the incidence of late (more than 100 days post HSCT) CMV diseases (31), and exposes those who will not develop CMV infection/reactivation to side effects. Excitingly, a newly approved prophylactic anti-CMV drug, letermovir, can reduce the CMV infection rate from 60.6% (placebo group) to 37.5% within 24 weeks of follow-up in seropositive HSCT settings, with tolerable safety profiles (32). It is also possibly beneficial for some CAR-T recipients. Routine detection of CMV DNA in the blood is recommended, which provides signals to initiate pre-emptive therapy, and therefore effectively prevents CMV diseases (33). However, there are studies arguing that it is not necessary to monitor CMV infection/reactivation in patients other than allo-HSCT recipients since only a small proportion of patients with CMV DNAemia develop into CMV disease (34, 35). Nevertheless, CAR-T cell therapy creates a totally novel situation that is different from those associated with other therapies.

The primary pre-emptive treatment of CMV infection/reactivation is the intravenous ganciclovir or foscarnet, or oral valganciclovir, with a duration of at least 2 weeks. The viral load should be monitored during therapy, which is important for the therapeutic instruction, but regretfully was not performed in this case. The intravenous transfusion of immunoglobulin or CMV antibodies is used in clinical practice as an adjuvant therapy, with no consistent evidence supporting the effectiveness. The main obstacles to administering these regimens are safety concerns and drug resistance (36). In this case, the myelosuppression side-effect of anti-viral medicine (ganciclovir/penciclovir), together with the potential reduced bone marrow reserve after CAR-T therapy and CMV infection itself, led to the dramatic decrease in CBC and intolerance to the therapy. The second-line therapy includes a combination of half-dose of foscarnet and ganciclovir, and cidofovir (37, 38). Novel therapies such as adoptive CMV-specific T cell transfusion are being researched, with promising primary outcomes (39, 40). A similar regimen is commended for CMV pneumonia. Altogether, a speedy recognition of CMV infection/reactivation and initiation of effective treatment is vitally important for patients with a likelihood of developing CMV end-organ disease.

In conclusion, we report a case of rare fatal CMV pneumonia three months after CAR-T cell therapy to raise awareness of the issue of severe CMV disease after CAR-T therapy. Additionally, lots of CAR-T recipients have experienced allo-HSCT, which complicates the problems of CMV reactivation after CAR-T infusion. Thus, during the outpatient follow-up of these patients, close attention should be paid to CMV reactivation. It would be interesting to investigate the benefit of prophylactic therapy using letermovir in the clinical setting, and estimate the high-risk population to develop CMV reactivation and CMV disease.

## Patient perspective

The patient and his parents were fully engaged throughout the treatment process. We’ve had thorough discussions with them about the CAR-T therapy, the diagnosis, treatment and prognosis of CMV pneumonia and the original disease in a timely manner, and collectively reached a therapeutic decision. We obtained the informed consent to report this case from the patient’s father.

## Data availability statement

The original contributions presented in the study are included in the article/supplementary material. Further inquiries can be directed to the corresponding author.

## Ethics statement

The studies involving humans were approved by Medical Ethics Committee of the Department of Hematology, Tongji Hospital, Tongji Medical College, Huazhong University of Science and Technology (TJ-IRB20160310). The studies were conducted in accordance with the local legislation and institutional requirements. Written informed consent for participation in this study was provided by the participants’ legal guardians/next of kin. Written informed consent was obtained from the individual(s) for the publication of any potentially identifiable images or data included in this article. Written informed consent was obtained from the participant/patient(s) for the publication of this case report.

## Author contributions

JC analyzed the data and wrote the manuscript. XM conducted the flow cytometry. LC conducted the ddPCR. JH, WC, LH and JZ managed the patients. NW revised the manuscript and oversaw the final approval of the manuscript. All authors contributed to the article and approved the submitted version.

## References

[1] Maude SL Frey N Shaw PA Aplenc R Barrett DM Bunin NJ. Chimeric antigen receptor T cells for sustained remissions in leukemia. N Engl J Med (2014) 371(16):1507–17. doi: 10.1056/NEJMoa1407222 PMC4267531 25317870

[2] Locke FL Neelapu SS Bartlett NL Siddiqi T Chavez JC Hosing CM. Phase 1 results of ZUMA-1: A multicenter study of KTE-C19 anti-CD19 CAR T cell therapy in refractory aggressive lymphoma. Mol Ther (2017) 25(1):285–95. doi: 10.1016/j.ymthe.2016.10.020 PMC5363293 28129122

[3] Raje N Berdeja J Lin Y Siegel D Jagannath S Madduri D. Anti-BCMA CAR T-cell therapy bb2121 in relapsed or refractory multiple myeloma. N Engl J Med (2019) 380(18):1726–37. doi: 10.1056/NEJMoa1817226 PMC8202968 31042825

[4] Hedrick SM. Chimeric T cell receptor-immunoglobulin molecules: function and applications. Int Rev Immunol (1993) 10(2-3):279–90. doi: 10.3109/08830189309061702 8360589

[5] Hill JA Li D Hay KA Green ML Cherian S Chen X. Infectious complications of CD19-targeted chimeric antigen receptor-modified T-cell immunotherapy. Blood (2018) 131(1):121–30. doi: 10.1182/blood-2017-07-793760 PMC5755046 29038338

[6] Wei J Zhu X Mao X Huang L Meng F Zhou J. Severe early hepatitis B reactivation in a patient receiving anti-CD19 and anti-CD22 CAR T cells for the treatment of diffuse large B-cell lymphoma. J Immunother Cancer (2019) 7(1):315. doi: 10.1186/s40425-019-0790-y 31753002 PMC6868854

[7] Li TD Li JJ Huang X Wang H Guo XY Ge SX. Baseline antibody level may help predict the risk of active human cytomegalovirus infection in a HCMV seropositive population. Eur J Clin Microbiol Infect Dis (2017) 36(5):863–8. doi: 10.1007/s10096-016-2873-8 28032284

[8] Ljungman P Boeckh M Hirsch HH Josephson F Lundgren J Nichols G. Definitions of cytomegalovirus infection and disease in transplant patients for use in clinical trials. Clin Infect Dis (2017) 64(1):87–91. doi: 10.1093/cid/ciw668 27682069

[9] Dioverti MV Razonable RR. Cytomegalovirus. Microbiol Spectr (2016) 4(4). doi: 10.1128/microbiolspec.DMIH2-0022-2015 27726793

[10] Cho SY Lee DG Kim HJ. Cytomegalovirus infections after hematopoietic stem cell transplantation: current status and future immunotherapy. Int J Mol Sci (2019) 20(11):2666. doi: 10.3390/ijms20112666 31151230 PMC6600658

[11] Ng AP Worth L Chen L Seymour JF Prince HM Slavin M. Cytomegalovirus DNAemia and disease: incidence, natural history and management in settings other than allogeneic stem cell transplantation. Haematologica (2005) 90(12):1672–9. 16330442

[12] Grønborg HL Jespersen S Hønge BL Jensen-Fangel S Wejse C. Review of cytomegalovirus coinfection in HIV-infected individuals in Africa. Rev Med Virol (2017) 27(1). doi: 10.1002/rmv.1907 27714898

[13] Chang H Tang TC Hung YS Lin TL Kuo MC Wang PN. Cytomegalovirus infection in non-transplant patients with hematologic neoplasms: a case series. Chang Gung Med J (2011) 34(1):65–74. 21392476

[14] [Chinese guidelines for diagnosis and treatment of acute lymphoblastic leukemia (2016)]. Zhonghua xue ye xue za zhi = Zhonghua xueyexue zazhi (2016) 37(10):837–45. doi: 10.3760/cma.j.issn.0253-2727.2016.10.002 PMC7364866 27801310

[15] Kantarjian H Thomas D O'Brien S Cortes J Giles F Jeha S. Long-term follow-up results of hyperfractionated cyclophosphamide, vincristine, doxorubicin, and dexamethasone (Hyper-CVAD), a dose-intensive regimen, in adult acute lymphocytic leukemia. Cancer (2004) 101(12):2788–801. doi: 10.1002/cncr.20668 15481055

[16] Xiao X He X Li Q Zhang H Meng J Jiang Y. Plasma exchange can be an alternative therapeutic modality for severe cytokine release syndrome after chimeric antigen receptor-T cell infusion: A case report. Clin Cancer Res (2019) 25(1):29–34. doi: 10.1158/1078-0432.CCR-18-1379 30322878

[17] Wudhikarn K Palomba ML Pennisi M Garcia-Recio M Flynn JR Devlin SM. Infection during the first year in patients treated with CD19 CAR T cells for diffuse large B cell lymphoma. Blood Cancer J (2020) 10(8):79. doi: 10.1038/s41408-020-00346-7 32759935 PMC7405315

[18] Vora SB Waghmare A Englund JA Qu P Gardner RA Hill JA. Infectious complications following CD19 chimeric antigen receptor T-cell therapy for children, adolescents, and young adults. Open Forum Infect Dis (2020) 7(5):ofaa121. doi: 10.1093/ofid/ofaa121 32432149 PMC7221263

[19] Park JH Romero FA Taur Y Sadelain M Brentjens RJ Hohl TM. Cytokine release syndrome grade as a predictive marker for infections in patients with relapsed or refractory B-cell acute lymphoblastic leukemia treated with chimeric antigen receptor T cells. Clin Infect Dis (2018) 67(4):533–40. doi: 10.1093/cid/ciy152 PMC6070095 29481659

[20] Ljungman P de la Camara R Robin C Crocchiolo R Einsele H Hill JA. Guidelines for the management of cytomegalovirus infection in patients with haematological Malignancies and after stem cell transplantation from the 2017 European Conference on Infections in Leukaemia (ECIL 7). Lancet Infect Dis (2019) 19(8):e260–72. doi: 10.1016/S1473-3099(19)30107-0 31153807

[21] Chemaly RF Torres HA Hachem RY Nogueras GM Aguilera EA Younes A. Cytomegalovirus pneumonia in patients with lymphoma. Cancer (2005) 104(6):1213–20. doi: 10.1002/cncr.21294 16078263

[22] Sylwester AW Mitchell BL Edgar JB Taormina C Pelte C Ruchti F. Broadly targeted human cytomegalovirus-specific CD4+ and CD8+ T cells dominate the memory compartments of exposed subjects. J Exp Med (2005) 202(5):673–85. doi: 10.1084/jem.20050882 PMC2212883 16147978

[23] Martins JP Andoniou CE Fleming P Kuns RD Schuster IS Voigt V. Strain-specific antibody therapy prevents cytomegalovirus reactivation after transplantation. Science (2019) 363(6424):288–93. doi: 10.1126/science.aat0066 30655443

[24] Han XY. Epidemiologic analysis of reactivated cytomegalovirus antigenemia in patients with cancer. J Clin Microbiol (2007) 45(4):1126–32. doi: 10.1128/JCM.01670-06 PMC1865821 17287334

[25] Nguyen Q Estey E Raad I Rolston K Kantarjian H Jacobson K. Cytomegalovirus pneumonia in adults with leukemia: an emerging problem. Clin Infect Dis (2001) 32(4):539–45. doi: 10.1086/318721 11181115

[26] Hakki M Aitken SL Danziger-Isakov L Michaels MG Carpenter PA Chemaly RF. American society for transplantation and cellular therapy series: #3-prevention of cytomegalovirus infection and disease after hematopoietic cell transplantation. Transplant Cell Ther (2021) 27(9):707–19. doi: 10.1016/j.jtct.2021.05.001 34452721

[27] Goldsmith SR Abid MB Auletta JJ Bashey A Beitinjaneh A Castillo P. Posttransplant cyclophosphamide is associated with increased cytomegalovirus infection: a CIBMTR analysis. Blood (2021) 137(23):3291–305. doi: 10.1182/blood.2020009362 PMC8351903 33657221

[28] Nichols WG Price TH Gooley T Corey L Boeckh M. Transfusion-transmitted cytomegalovirus infection after receipt of leukoreduced blood products. Blood (2003) 101(10):4195–200. doi: 10.1182/blood-2002-10-3143 12531791

[29] Ljungman P de La Camara R Milpied N Volin L Russell CA Crisp A. Randomized study of valacyclovir as prophylaxis against cytomegalovirus reactivation in recipients of allogeneic bone marrow transplants. Blood (2002) 99(8):3050–6. doi: 10.1182/blood.V99.8.3050 11929799

[30] Goodrich JM Bowden RA Fisher L Keller C Schoch G Meyers JD. Ganciclovir prophylaxis to prevent cytomegalovirus disease after allogeneic marrow transplant. Ann Intern Med (1993) 118(3):173–8. doi: 10.7326/0003-4819-118-3-199302010-00003 8380242

[31] Boeckh M Ljungman P. How we treat cytomegalovirus in hematopoietic cell transplant recipients. Blood (2009) 113(23):5711–9. doi: 10.1182/blood-2008-10-143560 PMC2700312 19299333

[32] El Helou G Razonable RR. Letermovir for the prevention of cytomegalovirus infection and disease in transplant recipients: an evidence-based review. Infect Drug Resist (2019) 12:1481–91. doi: 10.2147/IDR.S180908 PMC6556539 31239725

[33] Chen K Cheng MP Hammond SP Einsele H Marty FM. Antiviral prophylaxis for cytomegalovirus infection in allogeneic hematopoietic cell transplantation. Blood Adv (2018) 2(16):2159–75. doi: 10.1182/bloodadvances.2018016493 PMC6113617 30154125

[34] Sandherr M Hentrich M von Lilienfeld-Toal M Massenkeil G Neumann S Penack O. Antiviral prophylaxis in patients with solid tumours and haematological Malignancies–update of the Guidelines of the Infectious Diseases Working Party (AGIHO) of the German Society for Hematology and Medical Oncology (DGHO). Ann Hematol (2015) 94(9):1441–50. doi: 10.1007/s00277-015-2447-3 PMC4525190 26193852

[35] Marchesi F Pimpinelli F Ensoli F Mengarelli A. Cytomegalovirus infection in hematologic Malignancy settings other than the allogeneic transplant. Hematol Oncol (2018) 36(2):381–91. doi: 10.1002/hon.2453 28660653

[36] El Chaer F Shah DP Chemaly RF. How I treat resistant cytomegalovirus infection in hematopoietic cell transplantation recipients. Blood (2016) 128(23):2624–36. doi: 10.1182/blood-2016-06-688432 PMC5146744 27760756

[37] Mattes FM Hainsworth EG Geretti AM Nebbia G Prentice G Potter M. A randomized, controlled trial comparing ganciclovir to ganciclovir plus foscarnet (each at half dose) for preemptive therapy of cytomegalovirus infection in transplant recipients. J Infect Dis (2004) 189(8):1355–61. doi: 10.1086/383040 15073671

[38] Ljungman P Deliliers GL Platzbecker U Matthes-Martin S Bacigalupo A Einsele H. Cidofovir for cytomegalovirus infection and disease in allogeneic stem cell transplant recipients. The Infectious Diseases Working Party of the European Group for Blood and Marrow Transplantation. Blood (2001) 97(2):388–92. doi: 10.1182/blood.V97.2.388 11154213

[39] Holmes-Liew CL Holmes M Beagley L Hopkins P Chambers D Smith C. Adoptive T-cell immunotherapy for ganciclovir-resistant CMV disease after lung transplantation. Clin Transl Immunol (2015) 4(3):e35. doi: 10.1038/cti.2015.5 PMC4386617 25859390

[40] Tzannou I Papadopoulou A Naik S Leung K Martinez CA Ramos CA. Off-the-shelf virus-specific T cells to treat BK virus, human herpesvirus 6, cytomegalovirus, Epstein-Barr virus, and adenovirus infections after allogeneic hematopoietic stem-cell transplantation. J Clin Oncol (2017) 35(31):3547–57. doi: 10.1200/JCO.2017.73.0655 PMC5662844 28783452

